# Association between anion gap and postoperative delirium in patients undergoing open heart surgery

**DOI:** 10.3389/fcvm.2025.1592161

**Published:** 2025-05-19

**Authors:** Jun Wang, Hui Zhong, Lu Chen, Hong-Chun Ding, Zhong-Jie Lu, Bin-Su Wang, Shun-Bi Liu, Jing Luo, Li-Wen Hou, Yuan-Zhang Liu, Sheng Ding, Feng Gao, Li Jiang

**Affiliations:** ^1^ ICU, Department of Cardiovascular Surgery, People’s Liberation Army The General Hospital of Western Theater Command, Chengdu, Sichuan, China; ^2^ Department of Cardiology, The Second Affiliated Hospital (Xinqiao Hospital) of Army Medical University, Chongqing, China

**Keywords:** anion gap, postoperative delirium, open heart surgery, intensive care unit, MIMIC-IV

## Abstract

**Background:**

Open heart surgery (OHS) is crucial for treating cardiovascular diseases, but postoperative delirium (POD) is a common and challenging complication. Existing POD prognostic indicators have limitations in clinical application. The relationship between AG and POD in OHS patients remains unclear.

**Methods:**

Data from the MIMIC-IV database were used. Patients aged 18 or older who underwent OHS, were admitted to the ICU post-surgery, and had an AG test within the first 24 h after surgery were included. The maximum AG value within 24 h after surgery was the exposure variable, and POD occurrence was the primary outcome. Multivariable logistic regression was applied to explore the relationship between AG and POD. A restricted cubic spline regression model (RCSRM) was used to analyze the correlation shape, and subgroup and interaction analyses were performed. Causal mediation analysis (CMA) was conducted to explore the mediating role of ICU length of stay (LOS) in the relationship between AG and POD.

**Results:**

We included 6,429 patients. The overall POD incidence was 13%. Multivariable logistic regressions showed that AG was significantly associated with POD (OR = 1.686, 95% CI: 1.348–2.113, *P* < 0.001 for group 2; OR = 1.54, 95% CI: 1.161–2.037, *P* = 0.003 for group 3; OR = 2.005, 95% CI: 1.574–2.558, *P* < 0.001 for group 4; *P* for trend <0.001) and ICU LOS (OR = 1.256, 95% CI: 1.066–1.48, *P* = 0.007 for group 2; OR = 1.281, 95% CI: 1.033–1.585, *P* = 0.023 for group 3; OR = 1.595, 95% CI: 1.32–1.928, *P* < 0.001 for group 4). The RCSRM revealed a non-linear relationship between AG and POD (*P*-overall <0.001, *P* for non-linear = 0.042). No multiplicative or additive interactions were detected between AG and any subgroup. CMA indicated that ICU LOS mediated 5.392% (95% CI: 0.483%–11.98%; *P* = 0.034) of the effect of AG on POD.

**Conclusion:**

An elevated AG level within the first 24 h after OHS is significantly associated with an increased risk of POD, and the relationship shows a tendency toward non-linearity. ICU LOS may proportionally mediate the impact of AG on POD development.

## 1 Introduction

Open Heart Surgery (OHS) is a pivotal modality for treating cardiovascular diseases. Nevertheless, a plethora of perioperative complications can emerge, among which postoperative delirium (POD) ranks as one of the most frequently encountered, continuing to pose formidable challenges to healthcare providers (1, 2). Delirium, an acute cognitive disorder characterized by fluctuation in perception, attention, and arousal, profoundly influences patient prognosis, manifested through extended hospitalizations, elevated medical expenditures, increased mortality, and cognitive impairments (3, 4). Despite identifying numerous prognostic indicators for POD, such as pain, sleep disruptions, and an Acute Physiology And Chronic Health Evaluation II (APACHE II) score surpassing 20, most of these indicators are not readily amenable to widespread and facile application in clinical practice (1, 5–7). Hence, pursuing a straightforward and easily accessible clinical indicator for POD remains important for patients after OHS.

Hypoperfusion and acidosis are recognized as critical risk factors for POD. Acidosis, induced by tissue hypoxia and metabolic derangements, exerts a deleterious effect on the central nervous system by disrupting the equilibrium of neurotransmitters and impairs neuronal function, thereby culminating in delirium (8, 9). Moreover, hypoperfusion leads to a reduction in cerebral blood flow, further exacerbating brain injury and augmenting the risk of POD (10, 11). Serum anion gap (AG), a commonly utilized marker of acid-base balance in clinical settings, reflects the concentration differential between unmeasured anions and cations. Fluctuations in AG are intricately associated with the onset and progression of many diseases, including acidosis and hypoperfusion (12, 13). Hypoperfusion gives rise to tissue hypoxia, which, in turn, triggers cellular metabolic dysregulation and an upsurge in the production of acidic substances, thereby elevating AG (14, 15). Although prior studies have probed into the associations between AG and certain complications, such as mortality and acute kidney injury (AKI) (16–18), and have unearthed significant findings, a conspicuous research lacuna persists regarding the relationship between AG and POD in the cardiac surgery population.

This study endeavors to elucidate the association between AG and POD in patients undergoing OHS, thereby furnishing a foundation for the early clinical identification and prevention of POD.

## 2 Methods

### 2.1 Data source

The data were retrieved from the Medical Information Mart for Intensive Care IV (MIMIC-IV), which is administrated by the Beth Israel Deaconess Medical Center. The MIMIC-IV encompasses over 70,000 intensive care unit (ICU) admissions at the Beth Israel Deaconess Medical Center in Boston, Massachusetts (19). One of the authors, Jun Wang, obtained access to the database and was accountable for data extraction (certification number: 10145962). The MIMIC-IV database had been approved by the Institutional Review Boards (IRB) of the Massachusetts Institute of Technology and the Beth Israel Deaconess Medical Center, obviating the need for IRB approval from our institution. All data were extracted using structured query language. This study adheres to the STrengthening the Reporting of OBservational studies in Epidemiology (STROBE) statement for reporting (20).

### 2.2 Study population

Patients were eligible for inclusion if they met the following criteria: (1) aged 18 years or older; (2) underwent OHS, such as valvular surgery with extracorporeal circulation assistance or open-approach coronary artery bypass grafting (CABG); (3) were admitted to the ICU post-surgery; (4) had an AG test conducted within the first 24 h after surgery. For patients with multiple ICU admissions following surgery, only the first ICU stay was collected. Patients with an ICU length of stay (LOS) less than 24 h were excluded.

### 2.3 Exposure and outcomes

The exposure variable was defined as the maximum value of AG within the first 24 h after surgery. The primary outcome was the occurrence of POD after surgery. The incidence of POD was determined by a positive result of the Confusion Assessment Method for the ICU screen as recorded in the MIMIC-IV database. For patients who underwent multiple delirium assessments, only the first positive record was extracted; if all assessments were negative, only the first record was retrieved. The secondary outcomes included the development of AKI within the first 7 days after surgery, in-hospital mortality, and ICU LOS.

### 2.4 Covariates

Based on the study entry survey and reported confounding factors, several covariates were adjusted in the statistical analysis to mitigate potential confounding bias.

**Demographic information**: age, gender, body mass index (BMI), ethnicity.

**Severity Scores:** Sequential Organ Failure Assessment (SOFA), Simplified Acute Physiology Score II (SAPS II), Glasgow Coma Scale (GCS), Acute Physiology Score III (APS III), Charlson Comorbidity Index (CCI) (21–25).

**Comorbidity information:** congestive heart failure (CHF), cerebrovascular disease (CVD), chronic pulmonary disease (CPD), renal disease, severe liver disease (SLD), rheumatic disease, diabetes, malignancy.

**Vital signs:** heart rate (HR), mean blood pressure (MBP), respiratory rate (RR), peripheral oxygen saturation (SpO₂).

**Laboratory tests:** white blood cell count (WBC), hematocrit, platelet count, serum glucose, chloride, sodium, potassium, international normalized ratio (INR).

**Special treatments during the first 24 h of ICU admission:** renal replacement therapy (RRT), invasive ventilation.

**Other information:** urine output during the first 24 h of ICU admission, surgery type.

### 2.5 Statistical methods

Multiple imputation was employed to circumvent bias stemming from missing data (26), and multivariable logistic regression was utilized to control for confounding effects attributable to differences in baseline characteristics among groups and to elucidate the association between AG and POD. Baseline characteristics were presented using descriptive statistics. Kolmogorov–Smirnov test, Anderson–Darling test, and Lilliefors test were conducted to decide whether the variables follow a normal distribution. Continuous data are presented as means ± standard deviations or medians and interquartile ranges (IQRs) as appropriate. Kruskal–Wallis tests or analysis of variance (ANOVA), as appropriate for continuous variables, and chi-squared tests for categorical variables were used to compute differences in baseline characteristics among groups. A restricted cubic spline regression model (RCSRM) with 3 knots at the 10th, 50th, and 90th percentiles was utilized to depict the form of the correlation between AG and POD (27). Subgroup and interaction analyses were carried out to assess the robustness of our findings and to identify potential influencing factors. The synergy index (SI), the attributable proportion due to interaction (AP), and the relative excess risk due to interaction (RERI) were applied to evaluate the additive interactions. The effects of interaction analysis between AG and covariates were reported as odds ratios (ORs) and 95% confidence intervals (CIs). Causal mediation analysis (CMA) was conducted to explore whether the effect of AG on the incidence of POD was proportionally mediated by ICU LOS. Baseline variables were adjusted for in CMA. CMA is a method that decomposes the total effect of the exposure on the outcome into direct and indirect effects, with the latter being mediated by a mediator. This analysis yields the total effect, an average direct effect, and an average causal mediation effect (ACME) (28).

All statistical analyses were performed using R software (version 4.2.3, http://www.R-project.org/). Partial eta-squared of continuous variables and Cramer's *V* of categorical variables were computed to quantify the effect size among groups. Partial eta-squared is commonly used for continuous variables as it takes into account the variance explained by the group factor relative to the total variance, providing a more accurate measure of the practical significance of group differences. Cramer's *V* is appropriate for categorical variables as it assesses the strength of the association between two categorical variables in a multi-group context. A *P*-value <0.05 was considered statistically significant.

## 3 Results

### 3.1 Selection and clinical characteristics of the study population

**Figure 1:**
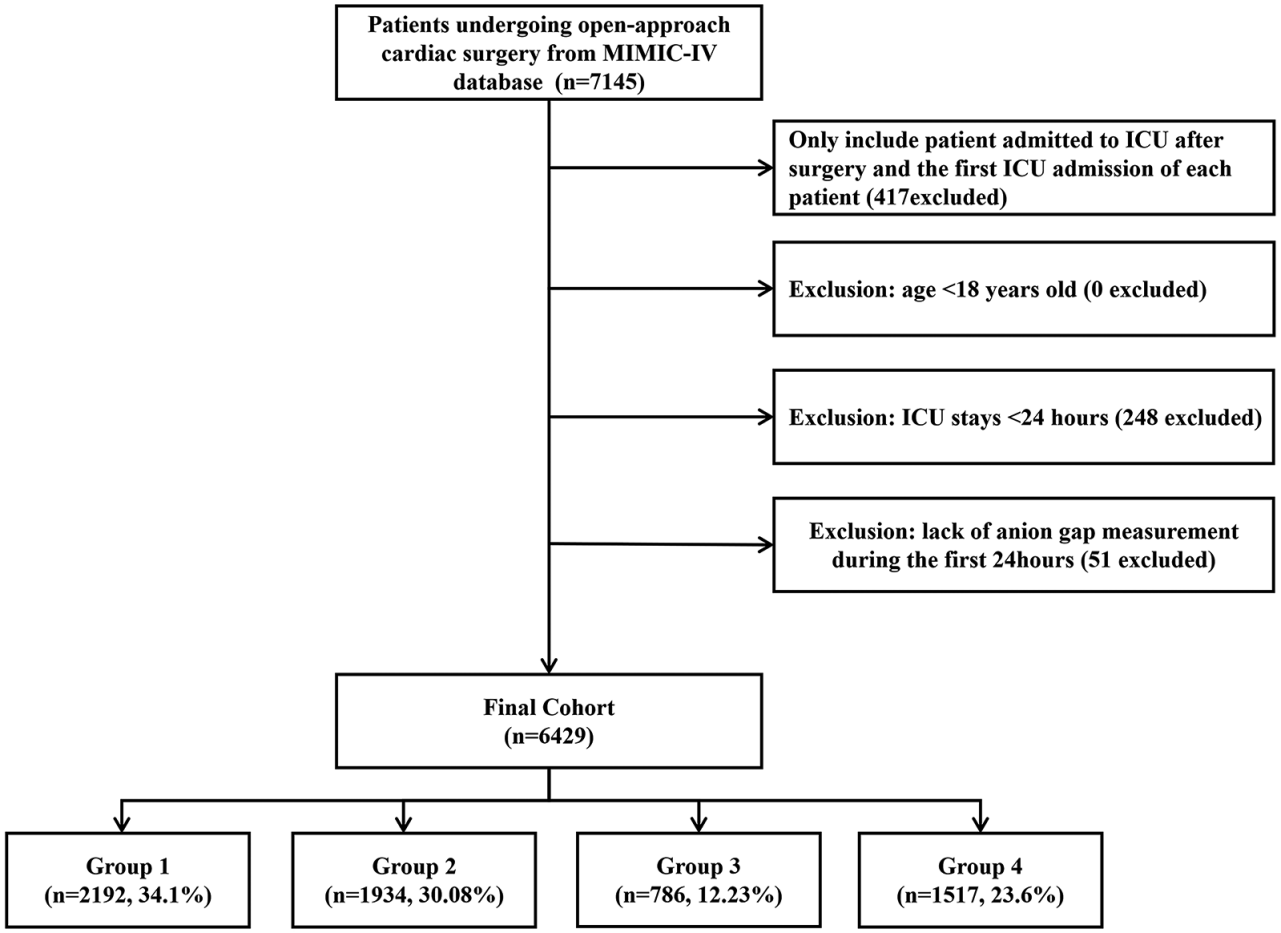
Flowchart of the study population.

Among 76,943 ICU admissions, 7,145 patients who underwent OHS were identified. After including only those admitted to the ICU after surgery and the first ICU admission for each patient, and excluding patients with an ICU stay of less than 24 h or those lacking an AG measurement within the first 24 h, a total of 6,429 patients were ultimately included in our study. The selection process is depicted in Figure 1.

The results of Normality test are presented in Supplementary Table 1. Of all the included patients, 4,690 (73%) were male, with a median age of 68.73 (IQR, 61.03–76.24) years. The median value of the AG was 13.00 (IQR, 11.00–14.00). The SOFA, SAPS II, and APS III of patients with a higher AG were greater than those in the first quartile (group 1). Patients in the second (group 2), third (group 3), and fourth (group 4) quartiles were more likely to have a history of CVDs. The BMIs of patients in group 2, group 3, and group 4 were higher than those in group 1 (Table 1).

**Table 1:** Characteristics of included patients according to the quartiles of serum AG levels.

Baseline characteristics	Overall	Group 1	Group 2	Group 3	Group 4	*P*	Effect size	Missing data (%)
*n*	6,429	2,192	1,934	786	1,517			
Age, years	68.73 (61.03, 76.24)	68.57 (60.85, 75.66)	69.01 (61.11, 76.41)	69.39 (61.15, 77.32)	68.57 (61.12, 76.34)	0.175	0.001	0
Male, *n* (%)	4,690 (73.0)	1,631 (74.4)	1,385 (71.6)	580 (73.8)	1,094 (72.1)	0.177	0.028	0
BMI, kg/m^2^	29.39 (26.06, 33.29)	28.98 (25.80, 32.36)	29.39 (25.99, 33.45)	29.32 (26.12, 33.27)	30.19 (26.56, 34.14)	<0.001	0.005	3.6
Ethnicity, *n* (%)						0.321	0.023	0
Asian	138 (2.1)	40 (1.8)	50 (2.6)	18 (2.3)	30 (2.0)			
Black	258 (4.0)	77 (3.5)	71 (3.7)	34 (4.3)	76 (5.0)			
Others	1,304 (20.3)	452 (20.6)	381 (19.7)	168 (21.4)	303 (20.0)			
White	4,729 (73.6)	1,623 (74.0)	1,432 (74.0)	566 (72.0)	1,108 (73.0)			
Severity scores								
SOFA	5.00 (4.00, 8.00)	5.00 (4.00, 7.00)	5.00 (4.00, 7.00)	5.50 (4.00, 8.00)	6.00 (4.00, 9.00)	<0.001	0.056	0
SAPS II	36.00 (29.00, 43.00)	34.00 (28.00, 42.00)	35.00 (29.00, 42.00)	36.00 (30.00, 43.00)	38.00 (31.00, 48.00)	<0.001	0.022	0
GCS	14.00 (13.00, 15.00)	14.00 (14.00, 15.00)	14.00 (14.00, 15.00)	14.00 (13.00, 15.00)	14.00 (12.00, 15.00)	<0.001	0.004	0
APS III	35.00 (27.00, 48.00)	33.00 (26.00, 42.00)	34.00 (27.00, 45.75)	36.00 (28.00, 48.00)	41.00 (30.00, 60.00)	<0.001	0.036	0
CCI	5.00 (4.00, 6.00)	5.00 (4.00, 6.00)	5.00 (4.00, 6.00)	5.00 (4.00, 6.00)	6.00 (4.00, 7.00)	<0.001	0.026	0
Comorbidities, *n* (%)								
CHF	1,727 (26.9)	461 (21.0)	481 (24.9)	224 (28.5)	561 (37.0)	<0.001	0.138	0
CVD	658 (10.2)	206 (9.4)	201 (10.4)	79 (10.1)	172 (11.3)	0.288	0.024	0
CPD	1,413 (22.0)	471 (21.5)	449 (23.2)	150 (19.1)	343 (22.6)	0.101	0.031	0
Renal disease	1,095 (17.0)	241 (11.0)	313 (16.2)	142 (18.1)	399 (26.3)	<0.001	0.153	0
SLD	18 (0.3)	0 (0.0)	7 (0.4)	3 (0.4)	8 (0.5)	0.017	0.04	0
Rheumatic disease	197 (3.1)	56 (2.6)	68 (3.5)	18 (2.3)	55 (3.6)	0.092	0.032	0
Diabetes	2,399 (37.3)	733 (33.4)	708 (36.6)	294 (37.4)	664 (43.8)	<0.001	0.08	0
Malignant	181 (2.8)	71 (3.2)	53 (2.7)	18 (2.3)	39 (2.6)	0.457	0.02	0
Vital signs								
HR, bpm	81.69 (76.72, 87.79)	81.67 (77.13, 87.64)	81.47 (76.50, 87.52)	81.42 (76.53, 87.79)	82.10 (76.69, 88.47)	0.253	0.001	0
MBP, mmHg	73.72 (70.12, 77.25)	73.65 (70.30, 77.08)	73.93 (70.30, 77.48)	73.58 (70.05, 77.34)	73.52 (69.62, 77.29)	0.131	0.001	0
RR, bpm	17.37 (15.88, 19.05)	17.06 (15.65, 18.77)	17.32 (15.87, 18.94)	17.59 (16.20, 19.23)	17.75 (16.19, 19.60)	<0.001	0.013	0
SpO_2_, %	98.04 (97.08, 98.90)	98.09 (97.18, 98.93)	97.97 (97.00, 98.87)	98.08 (97.14, 98.89)	97.96 (96.93, 98.89)	<0.001	0.004	0
UO (1st 24 h), ml	1,825.00 (1,345.00, 2,455.00)	1,943.50 (1,465.00, 2,585.75)	1,821.00 (1,363.50, 2,435.00)	1,835.00 (1,341.25, 2,435.00)	1,650.00 (1,120.00, 2,260.00)	<0.001	0.021	0.4
Laboratory tests								
WBC, 109/L	15.60 (12.50, 19.60)	14.80 (11.90, 18.40)	15.60 (12.60, 19.30)	15.90 (12.70, 19.88)	16.90 (13.30, 21.60)	<0.001	0.018	0
Hematocrit, %	26.30 (23.60, 29.50)	26.30 (23.70, 29.40)	26.50 (23.80, 29.70)	26.65 (23.50, 29.90)	25.90 (23.10, 29.30)	0.003	0.002	0
Platelets, 109/L	129.00 (104.00, 161.00)	130.00 (106.00, 159.00)	131.00 (104.00, 163.00)	129.00 (103.00, 162.00)	126.00 (101.00, 160.00)	0.154	0.001	0
Serum glucose, mg/dl	121.00 (106.00, 139.00)	115.00 (100.00, 131.00)	120.00 (105.00, 136.00)	123.00 (109.25, 140.00)	130.00 (113.00, 158.00)	<0.001	0.055	1.8
Chloride, mmol/L	109.00 (107.00, 112.00)	110.00 (108.00, 112.00)	109.00 (107.00, 112.00)	109.00 (107.00, 111.00)	108.00 (106.00, 110.00)	<0.001	0.072	0
Sodium, mmol/L	140.00 (138.00, 141.00)	139.00 (137.00, 141.00)	140.00 (138.00, 141.00)	140.00 (138.00, 141.00)	140.00 (138.00, 142.00)	<0.001	0.011	0
Potassium, mmol/L	4.60 (4.40, 4.90)	4.60 (4.30, 4.80)	4.60 (4.40, 4.90)	4.60 (4.40, 4.90)	4.70 (4.40, 5.00)	<0.001	0.009	0.2
INR	1.40 (1.30, 1.60)	1.40 (1.30, 1.50)	1.40 (1.30, 1.60)	1.40 (1.30, 1.60)	1.50 (1.30, 1.70)	<0.001	0.014	0
Interventions (1st 24 h), *n* (%)								
RRT	60 (0.9)	4 (0.2)	3 (0.2)	4 (0.5)	49 (3.2)	<0.001	0.133	0
Invasive ventilation	4,271 (66.4)	1,408 (64.2)	1,247 (64.5)	531 (67.6)	1,085 (71.5)	<0.001	0.064	0
Surgery type, *n* (%)						<0.001	0.103	0
CABG	2,252 (35.0)	655 (29.9)	595 (30.8)	297 (37.8)	705 (46.5)			
Others	2,241 (34.9)	846 (38.6)	690 (35.7)	273 (34.7)	432 (28.5)			
Valve	1,936 (30.1)	691 (31.5)	649 (33.6)	216 (27.5)	380 (25.0)			
AG, mmol/L	13.00 (11.00, 14.00)	10.00 (9.00, 11.00)	12.00 (12.00, 13.00)	14.00 (14.00, 14.00)	16.00 (15.00, 17.00)	<0.001	0.78	0

AG, anion gap; BMI, body mass index; SOFA, Sequential Organ Failure Assessment; SAPS II, Simplified Acute Physiology Score II, GCS, Glasgow Coma Scale; APS III, Acute Physiology Score III; CHF, Congestive heart failure; CVD, Cerebrovascular disease; CPD, Chronic pulmonary disease; SLD; severe liver disease; CCI, Charlson comorbidity index; HR, heart rate; MBP, mean blood pressure; RR, respiratory rate; SpO2, peripheral oxygen saturation; Urine output, UO; WBC, white blood cell; INR, international normalized ratio; RRT, renal replacement therapy; CABG, coronary artery bypass grafting.

### 3.2 Associations between AG and the incidence of POD

The overall incidence of POD was 834 (13%). When AG was treated as a continuous variable, logistic regression analysis demonstrated a significant association between AG and the incidence of POD (OR = 1.092, 95% CI: 1.058–1.127, *P* < 0.001). When AG was treated as categorical data, univariable logistic regression analysis revealed a significant association between AG and the incidence of POD (OR = 1.842, 95% CI: 1.496–2.275, *P* < 0.001 for group 2; OR = 2.023, 95% CI: 1.558–2.618, *P* < 0.001 for group 3; OR = 3.409, 95% CI: 2.788–4.182, *P* < 0.001 for group 4). This association remained robust after adjusting for all covariates (OR = 1.686, 95% CI: 1.348–2.113, *P* < 0.001 for group 2; OR = 1.54, 95% CI: 1.161–2.037, *P* = 0.003 for group 3; OR = 2.005, 95% CI: 1.574–2.558, *P* < 0.001 for group 4). The trend test was statistically significant (*P* for trend <0.001) (Table 2).

**Table 2:** Association between AG and the incidence of POD.

Serum AG level	Cases, percentile (95% CI)	Crude		Model 1		Model 2	
		OR (95% CI)	*P*	OR (95% CI)	*P*	OR (95% CI)	*P*
Total	834, 13% (12.2%–13.8%)	—	—	—	—	—	—
Group 1	160, 7.3% (6.2%–8.5%)	ref	ref	ref	ref	ref	ref
Group 2	245, 12.7% (11.2%–14.2%)	1.842 (1.496, 2.275)	<0.001	1.804 (1.463, 2.23)	<0.001	1.686 (1.348, 2.113)	<0.001
Group 3	108, 13.7% (11.4%–16.3%)	2.023 (1.558, 2.618)	<0.001	1.979 (1.522, 2.565)	<0.001	1.54 (1.161, 2.037)	0.003
Group 4	321, 21.2% (19.1%–23.3%)	3.409 (2.788, 4.182)	<0.001	3.404 (2.78, 4.182)	<0.001	2.005 (1.574, 2.558)	<0.001
*P* for trend	—	<0.001	<0.001	<0.001			
AG <13 mmol/L*	271, 8.6% (7.6%–9.6%)	ref	ref	ref	ref	ref	ref
AG ≥13 mmol/L*	563, 17.3% (16%–18.6%)	2.232 (1.915–2.607)	<0.001	2.223 (1.906–2.599)	<0.001	1.535 (1.286–1.835)	<0.001
Ag as a continuous variable	—	1.174 (1.147, 1.202)	<0.001	1.179 (1.152, 1.207)	<0.001	1.092 (1.058, 1.127)	<0.001

Mode 1, adjusted for gender and age; Model 2, adjusted for age, gender, BMI, ethnicity, SOFA, SAPS II, GCS, APS III, CCI, CHF, CVD, CPD, renal disease, SLD, rheumatic disease, diabetes, malignancy, HR, MBP, RR, SpO2, UO, WBC, hematocrit, platelets, serum glucose, chloride, sodium, potassium, INR, surgery type, and interventions during the first 24 h (RRT, invasive ventilation).

* The cut-off value was 13 mmol/L according to the restricted cubic spline regression model.

AG, anion gap; POD, postoperative delirium; BMI, body mass index; SOFA, Sequential Organ Failure Assessment; SAPS II, Simplified Acute Physiology Score II, GCS, Glasgow Coma Scale; APS III, Acute Physiology Score III; CHF, Congestive heart failure; CVD, Cerebrovascular disease; CPD, Chronic pulmonary disease; SLD; severe liver disease; CCI, Charlson comorbidity index; HR, heart rate; MBP, mean blood pressure; RR, respiratory rate; SpO2, peripheral oxygen saturation; Urine output, UO; WBC, white blood cell; INR, international normalized ratio; RRT, renal replacement therapy; OR, odds ratio; CI, confidence interval.

**Figure 2:**
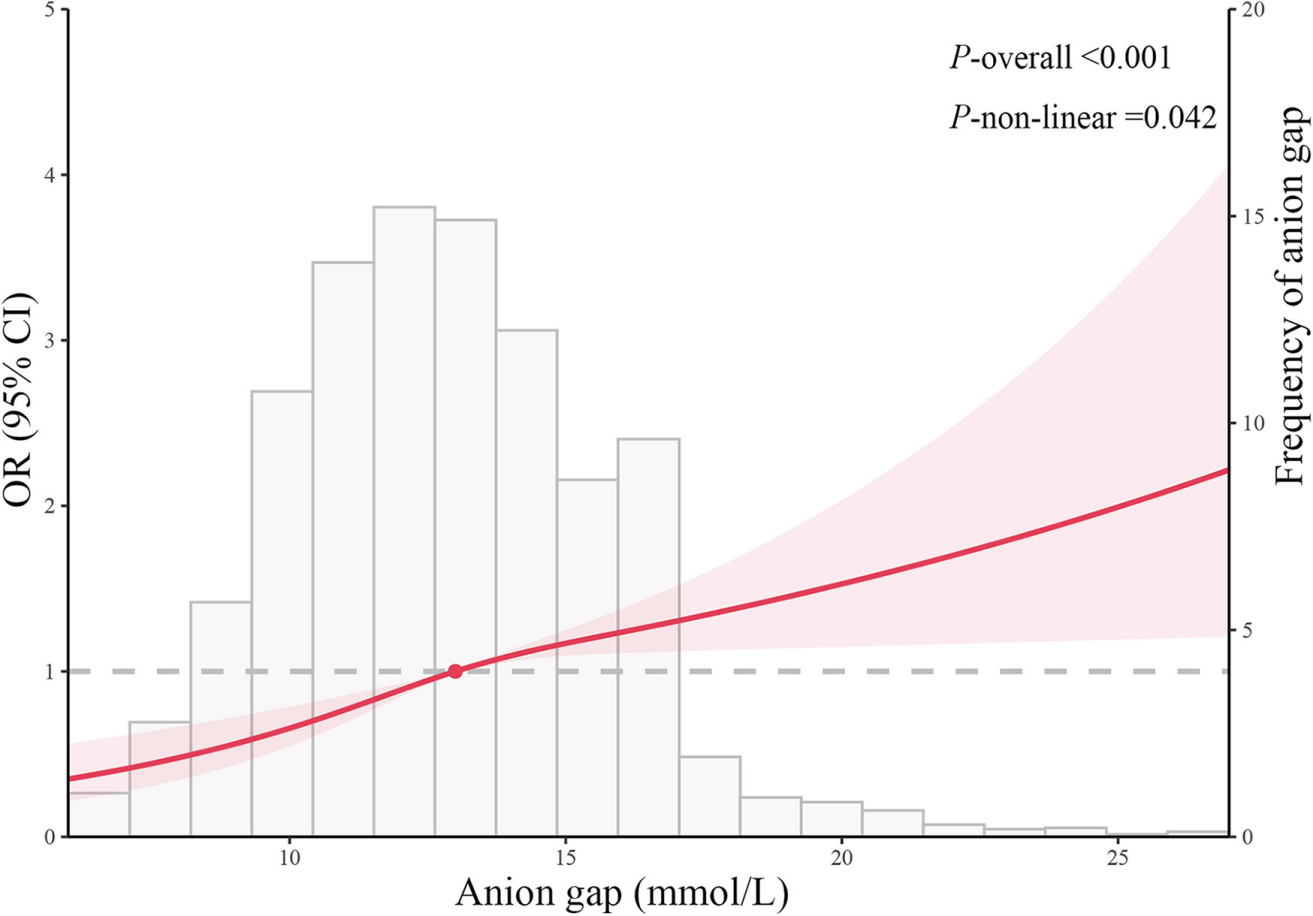
Cubic model of the correlation between anion gap and postoperative delirium after adjusting for baseline variables.

After adjusting for all covariates, the RCSRM revealed a non-linear correlation between AG and the incidence of POD (*P*-overall <0.001, *P* for non-linear = 0.042) (Figure 2). The RCSRM showed that the cut-off value of AG was 13 mmol/L. After grouping the patients based on this cut-off value and performing logistic regression analysis, the results demonstrated that the correlation between AG and POD remained robust (OR 1.535, 95% CI: 1.286–1.835, *P* < 0.001) (Table 2).

### 3.3 Associations between AG and secondary outcomes

In the general cohort, 1,922 (29.9%) patients had an ICU LOS exceeding 3 days. Multivariable logistic regression analysis showed that AG was significantly associated with an ICU LOS of more than 3 days (OR = 1.256, 95% CI: 1.066–1.48, *P* = 0.007 for group 2; OR = 1.281, 95% CI: 1.033–1.585, *P* = 0.023 for group 3; OR = 1.595, 95% CI: 1.32–1.928, *P* < 0.001 for group 4). Logistic regression analyses indicated that AG was not associated with the development of AKI within the first 7 days after surgery or in-hospital mortality (Table 3).

**Table 3:** Association between AG and the incidence of secondary outcomes.

Secondary outcomes	Metrics	Total	Group 1	Group 2	Group 3	Group 4
AKI	Cases, percentile (95% CI)	5,001, 77.8% (76.8%–78.8%)	1,601, 73% (71.1%–74.9%)	1,520, 78.6% (76.7%–80.4%)	599, 76.2% (73.1%–79.1%)	1,281, 84.4% (82.5%–86.2%)
	OR (95% CI)	—	ref	1.104 (0.932, 1.308)	0.898 (0.716, 1.127)	1.139 (0.922, 1.409)
	*P*	—	ref	0.253	0.35	0.228
	*P* for trend		0.498			
In-hospital mortality*	Cases, percentile (95% CI)	92, 1.4% (1.2%–1.8%)	8, 0.4% (0.2%–0.7%)	14, 0.7% (0.4%–1.2%)	7, 0.9% (0.4%–1.8%)	63, 4.2% (3.2%–5.3%)
	OR (95% CI)	—	ref	1.227 (0.493, 3.226)	1.254 (0.413, 3.709)	1.709 (0.721, 4.411)
	*P*	—	ref	0.665	0.681	0.241
	*P* for trend		0.186			
ICU LOS	Cases, percentile (95% CI) (≥3 days)	1,922, 29.9% (28.8%–31%)	479, 21.9% (20.1%–23.6%)	544, 28.1%(26.1%–30.2%)	246, 31.3% (28.1%–34.7%)	653, 43% (40.5%–45.6%)
	OR (95% CI)	—	ref	1.256 (1.066, 1.48)	1.281 (1.033, 1.585)	1.595 (1.32, 1.928)
	*P* values of logistic regressions	—	ref	0.007	0.023	<0.001
	*P* for trend		<0.001			
	ICU LOS as continuous variable	1.99 (1.25, 3.21)	1.43 (1.21, 2.40)	1.81 (1.25, 3.14)	2.03 (1.26, 3.26)	2.33 (1.34, 4.23)

All the logistic regression models are adjusted for age, gender, BMI, ethnicity, SOFA, SAPS II, GCS, APS III, CCI, CHF, CVD, CPD, renal disease, SLD, rheumatic disease, diabetes, malignancy, HR, MBP, RR, SpO2, UO, WBC, hematocrit, platelets, serum glucose, chloride, sodium, potassium, INR, surgery type, and interventions during the first 24 h (RRT, invasive ventilation).

* Firth-corrected logistic regression.

AG, anion gap; POD, postoperative delirium; BMI, body mass index; SOFA, Sequential Organ Failure Assessment; SAPS II, Simplified Acute Physiology Score II, GCS, Glasgow Coma Scale; APS III, Acute Physiology Score III; CHF, Congestive heart failure; CVD, Cerebrovascular disease; CPD, Chronic pulmonary disease; SLD; severe liver disease; CCI, Charlson comorbidity index; HR, heart rate; MBP, mean blood pressure; RR, respiratory rate; SpO2, peripheral oxygen saturation; Urine output, UO; WBC, white blood cell; INR, international normalized ratio; RRT, renal replacement therapy; AKI, acute kidney injury; ICU LOS, length of stay in the intensive care unit; OR, odds ratio; CI, confidence interval.

### 3.4 Subgroup and interaction analyses

We conducted subgroup analyses to stratify the relationship between AG and the incidence of POD. The ORs of patients with CVDs were higher than those of patients without CVDs in group 2, group 3, and group 4. The ORs of patients with a BMI lower than 24 were higher than those of patients with a BMI of 24 or above in group 2, group 3, and group 4 (Table 4).

**Table 4:** Association between AG and the incidence of POD in different subgroups.

Subgroup variables	Cases/total	Group 1	Group 2, OR (95% CI)	Group 3, OR (95% CI)	Group 4, OR (95% CI)
Age, years					
≥60	712/5,007	Ref	1.597 (1.255, 2.038)	1.62 (1.196, 2.184)	1.975 (1.522, 2.569)
<60	122/1,422	Ref	2.725 (1.468, 5.249)	1.415 (0.615, 3.186)	2.07 (1.041, 4.217)
Gender					
Male	566/4,690	Ref	1.721 (1.318, 2.253)	1.384 (0.982, 1.938)	1.88 (1.411, 2.511)
Female	268/1,739	Ref	1.616 (1.062, 2.48)	1.918 (1.138, 3.214)	2.39 (1.506, 3.822)
BMI, kg/m^2^					
≥24	731/5,671	Ref	1.663 (1.31, 2.117)	1.41 (1.039, 1.905)	1.933 (1.494, 2.505)
<24	103/758	Ref	1.903 (0.963, 3.874)	2.428 (1.064, 5.538)	2.882 (1.333, 6.375)
Invasive ventilation					
Yes	701/4,271	Ref	1.629 (1.265, 2.104)	1.462 (1.064, 2)	2.051 (1.566, 2.692)
No	133/2,158	Ref	2 (1.219, 3.334)	1.741 (0.894, 3.307)	1.855 (1.034, 3.34)
CVD					
Yes	137/658	Ref	2.679 (1.462, 5.03)	1.76 (0.771, 3.92)	2.504 (1.266, 5.028)
No	697/5,771	Ref	1.56 (1.224, 1.994)	1.509 (1.114, 2.036)	1.931 (1.489, 2.509)
Surgery type					
CABG	387/2,252	Ref	1.356 (0.956, 1.927)	1.349 (0.882, 2.048)	1.413 (0.99, 2.023)
Others	189/2,241	Ref	2.026 (1.268, 3.284)	1.186 (0.616, 2.224)	3.195 (1.901, 5.438)
Valve	258/1,936	Ref	1.861 (1.253, 2.79)	2.201 (1.325, 3.638)	2.376 (1.483, 3.826)

Each subgroup analysis was adjusted (except for the subgroup variable) for age, gender, BMI, ethnicity, SOFA, SAPS II, GCS, APS III, CCI, CHF, CVD, CPD, renal disease, SLD, rheumatic disease, diabetes, malignancy, HR, MBP, RR, SpO2, UO, WBC, hematocrit, platelets, serum glucose, chloride, sodium, potassium, INR, surgery type, and interventions during the first 24 h (RRT, invasive ventilation). AG, anion gap; POD, postoperative delirium; BMI, body mass index; SOFA, Sequential Organ Failure Assessment; SAPS II, Simplified Acute Physiology Score II, GCS, Glasgow Coma Scale; APS III, Acute Physiology Score III; CHF, Congestive heart failure; CVD, Cerebrovascular disease; CPD, Chronic pulmonary disease; SLD; severe liver disease; CCI, Charlson comorbidity index; HR, heart rate; MBP, mean blood pressure; RR, respiratory rate; SpO2, peripheral oxygen saturation; Urine output, UO; WBC, white blood cell; INR, international normalized ratio; RRT, renal replacement therapy; OR, odds ratio; CI, confidence interval.

No multiplicative or additive interactions were detected between AG and any subgroup (Table 5). The synergy index (SI) of invasive ventilation and surgery type in the additive interaction analysis was not interpretable due to statistically meaningless values.

**Table 5:** Interaction analysis of subgroup variables and AG.

Variables	Multiplicative scale	Additive interaction		
		RERI	AP	SI
Age, years	0.941 (0.783, 1.13)	−0.011 (−0.554, 0.428)	−0.007 (−0.166, 0.206)	0.982 (0.789, 1.222)
Gender	0.947 (0.819, 1.096)	−0.061 (−0.348, 0.085)	−0.049 (−0.151, 0.115)	0.792 (0.558, 1.125)
BMI, kg/m^2^	0.847 (0.687, 1.044)	−0.115 (−0.837, 0.419)	−0.064 (−0.226, 0.133)	0.876 (0.746, 1.027)
Invasive ventilation	0.915 (0.771, 1.086)	−0.175 (−0.36, −0.1)	−0.33 (−0.623, −0.034)	NaN (NaN, NaN)
CVD	0.964 (0.798, 1.165)	0.009 (−0.476, 0.261)	0.006 (−0.116, 0.202)	1.017 (0.708, 1.459)
Surgery type	1.049 (0.968, 1.138)	−0.001 (−0.156, 0.043)	−0.001 (−0.069, 0.196)	NaN (NaN, NaN)

BMI, body mass index; CVD, cerebrovascular disease; RERI, relative excess risk due to interaction; AP, attributable proportion due to interaction; SI, synergy index.

### 3.5 CMA

**Figure 3:**
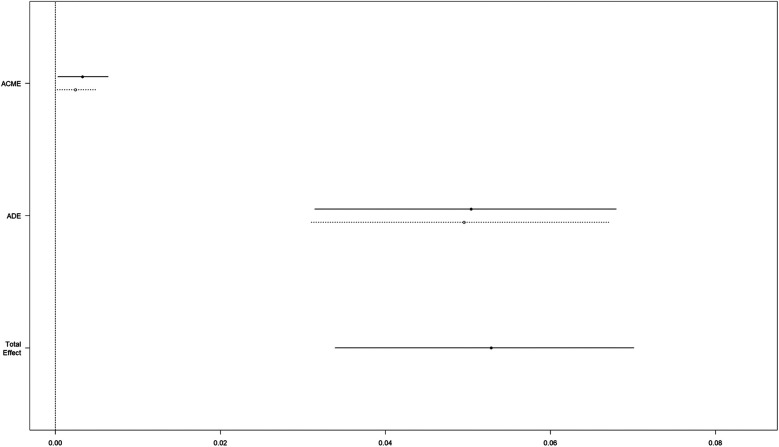
Causal mediation analysis for ICU LOS. The dashed line represents the group 1 group, and the solid line represents the other three groups (group 2, group 3, and group 4). ACME, average causal mediation effect; ADE, average direct effect; ICU LOS, length of stay in the intensive care unit; group 1, group 2, group 3, and group 4 represent the quartiles of the anion gap levels.

Regarding POD, CMA suggested that ICU LOS mediated 5.392% (95% CI: 0.483%–11.98%; *P* = 0.034) of the effect of AG (*P* = 0.034 for ACME) (Figure 3).

## 4 Discussion

Our study demonstrated that a higher AG level within the first 24 h after OHS was significantly associated with an increased risk of POD, and this relationship was non-linear.

The overall incidence of POD in our research was 13%, which is highly consistent with the findings of previous studies (29–33).

Serum AG reflects the status of electrolyte and acid-base balance. Previous studies have reported associations between electrolyte disorders/acid-base imbalances and POD in various surgical settings. For instance, the study by Li-Hong Wang et al. demonstrated the correlation between electrolyte disorders (hyponatremia and hypocalcemia) and POD after orthopedic surgery (34). Wei-Ying Zhang et al. found that electrolyte disorders were risk factors for POD after CABG (5). A meta-analysis by Shan Lu et al. revealed that electrolyte disorders were also risk factors for POD after Type-A aortic dissection surgery (6). Aldemir et al. confirmed that metabolic acidosis was a risk factor for POD in surgical ICU patients (35). The meta-analysis by Zaal et al. proved the correlation between metabolic acidosis and POD in non-cardiac surgery ICU patients (7).

However, none of these previous studies was able to establish the effect of AG on POD, let alone the shape of the relationship between AG and POD. Our study is the first to focus on the complex relationship between AG and POD, thus filling a partial gap in this field. Our findings underscore the clinical significance of an elevated anion gap (AG) as a biomarker indicating a heightened risk of postoperative delirium (POD). Therapeutic interventions, such as the correction of acidosis, may reduce the incidence of POD in patients undergoing OHS.

An elevated AG may indicate hypoxia, hypoperfusion, anemia, and ischemia, all of which are associated with an increased risk of delirium (1, 5, 7, 36). Our study also supports this, as the SpO₂ in the group 1 was higher than that in the other three groups. Acid-base and electrolyte disturbances are common in patients undergoing cardiac surgery. Hypoxia, ischemia, and acidosis frequently occur during the process of cardiopulmonary bypass, leading to an increase in acidic substances and AG levels. In this study, postoperative AG was associated with POD after adjusting for confounders. We also found that patients in the higher AG groups had higher SAPS II and APS-III scores, suggesting a possible association between a high AG and severe disease conditions. Therefore, AG may be related to postoperative ischemia and could potentially predict POD, although the underlying mechanism requires further investigation.

Additionally, an elevated AG may predict organ dysfunction. Yang et al. observed in their study that patients with a high AG had worse cardiac function and higher mortality (37). Zhang et al. reported that AG was a risk factor for mortality in ICU patients with heart diseases (16). Huang et al. demonstrated the relationship between elevated AG and mortality in patients with acute heart failure (38).In this study, we also found that patients in the higher AG groups more frequently had a history of CHF. Postoperative high AG may be a marker of cardiac dysfunction and predict a poorer prognosis. Moreover, we found that postoperative renal function was negatively correlated with the AG level, as patients in the higher AG groups had lower urine output. Up to 30% of patients develop AKI after cardiac surgery, increasing morbidity and mortality (39, 40). Zhang et al. reported the correlation between an increased AG and the incidence of AKI (18). Huang et al. reported the correlation between an increased AG and mortality (38). Similarly, in the present study, patients with a higher AG had a higher incidence of AKI within 7 days and in-hospital mortality, although these differences did not remain significant after adjusting for confounders, potentially due to differences in data sources and confounders between this study and previous ones.

The association between ICU LOS and POD has been reported in previous studies (1, 3, 4). In our study, we found that patients with a higher AG were more likely to have a longer ICU stay. The result of CMA further verified our hypothesis that ICU LOS plays a mediating role in the effect of AG on POD. That is, higher AG levels may prolong patients' ICU LOS, thereby contributing to the risk of POD. The relationship among AG, ICU LOS, and POD is complex. Elevated AG, often indicating metabolic acidosis, can trigger physiological changes that lead to more complex medical issues, necessitating longer ICU stays. During long ICU stays, patients are exposed to stressors like sleep disruption and invasive procedures, increasing POD risk.

Yujin Ko et al. reported that an underweight status is an independent risk factor for delirium in the ICU, while an obese or overweight status is not associated with delirium (41). Jianlei Fu et al. reported a U-shaped relationship between BMI and the incidence of delirium (42). In the subgroup analysis, we observed that the ORs of patients with a BMI lower than 24 were higher than those of patients with a BMI of 24 or above in group 2, group 3, and group 4, indicating a possible protective effect of being overweight in reducing the risk of POD. Nevertheless, no interaction was detected between AG and BMI concerning POD, suggesting that AG and BMI may exert their effects on POD independently.

This study also has certain limitations. First, the data were collected from a single medical institution, which may lead to insufficient representativeness and limit the generalizability of the research results. Multi-center and large-sample studies are needed to validate the universality of the conclusions of this study. Second, some unrecognized or difficult-to-measure factors might affect the incidence of POD, such as the patient's psychological stress state and pre-existing subclinical nervous system diseases, which, however, are not recorded in MIMIC-IV. Therefore, we included a set of confounding variables in the regression models to control bias induced by baseline characteristics as much as possible. Additionally, we used different statistical methods, including subgroup analyses, and RCSRM to test the robustness of our findings. Third, AG level might be affected by albumin level (43–45). Hence, albumin-corrected AG might exhibit a superior predictive capability compared to AG. But albumin-corrected AG was unsuitable in our cohort due to massive missing data of albumin, which might introduce bias into our results. Nevertheless, considering the limited existing research on the application of albumin-corrected AG in an OHS cohort and the lack of a well-defined relationship between albumin-corrected AG and uncorrected AG in such a cohort, a cautious attitude is advisable as the superiority of albumin-corrected AG over AG in an OHS cohort has not been conclusively established. Thus, we recommend conducting a comparison of the predictive capabilities between albumin-corrected AG and uncorrected AG within an OHS cohort in future research endeavors.

## Conclusion

An elevated AG level within the first 24 h following OHS is significantly associated with an increased risk of POD. The relationship between AG and POD shows a tendency toward non-linearity. Notably, ICU LOS may proportionally mediate the impact of AG on the development of POD after OHS.

## Data Availability

The datasets presented in this study can be found in online repositories. The names of the repository/repositories and accession number(s) can be found below: https://physionet.org/content/mimiciv/2.2/.
