# Supplementary material for: Association between anion gap and postoperative delirium in patients undergoing open heart surgery
Source: Front Cardiovasc Med. 2025 May 19;12:1592161. doi: 10.3389/fcvm.2025.1592161 (PMC12127371; doi:10.3389/fcvm.2025.1592161)
Supplement: Supplementary file 1 [file Table1.docx]

| Supplementary Table 1. Normality test of continuous variables | | | |
| --- | --- | --- | --- |
| Variables | *P* value | | |
|  | Kolmogorov-Smirnov test | Anderson-Darling test | Lilliefors test |
| Age | <0.001 | <0.001 | <0.001 |
| BMI | <0.001 | <0.001 | <0.001 |
| SOFA | <0.001 | <0.001 | <0.001 |
| SAPS II | <0.001 | <0.001 | <0.001 |
| GCS | <0.001 | <0.001 | <0.001 |
| APS III | <0.001 | <0.001 | <0.001 |
| CCI | <0.001 | <0.001 | <0.001 |
| HR | <0.001 | <0.001 | <0.001 |
| MBP | 0.051 | <0.001 | <0.001 |
| RR | <0.001 | <0.001 | <0.001 |
| SpO2 | <0.001 | <0.001 | <0.001 |
| UO (1st 24 hours) | <0.001 | <0.001 | <0.001 |
| WBC | <0.001 | <0.001 | <0.001 |
| Hematocrit | <0.001 | <0.001 | <0.001 |
| Platelets | <0.001 | <0.001 | <0.001 |
| Chloride | <0.001 | <0.001 | <0.001 |
| Serum glucose | <0.001 | <0.001 | <0.001 |
| Sodium | <0.001 | <0.001 | <0.001 |
| Potassium | <0.001 | <0.001 | <0.001 |
| AG | <0.001 | <0.001 | <0.001 |
| INR | <0.001 | <0.001 | <0.001 |
| BMI, body mass index; SOFA, Sequential Organ Failure Assessment; SAPS II, Simplified Acute Physiology Score II, GCS, Glasgow Coma Scale; APS III, Acute Physiology Score III; CCI, Charlson comorbidity index; HR, heart rate; MBP, mean blood pressure; RR, respiratory rate; SpO2, peripheral oxygen saturation; Urine output, UO; WBC, white blood cell; AG, anion gap; INR, international normalized ratio. | | | |
